# Accurately predicting heat transfer performance of ground-coupled heat pump system using improved autoregressive model

**DOI:** 10.7717/peerj-cs.482

**Published:** 2021-04-20

**Authors:** Zhaoyi Zhuang, Xinliang Zhai, Xianye Ben, Bin Wang, Dijia Yuan

**Affiliations:** 1 College of Thermal Energy Engineering, Shandong Jianzhu University, Jinan, China; 2 School of Electronic lnformation and Electrical Engineering, Shanghai Jiao Tong University, Shanghai, China; 3 School of Information Science and Engineering, Shandong University, Jinan, China

**Keywords:** Ground-coupled heat pump system, Autoregressive model with working condition inputs, Time series analysis, Random forests

## Abstract

Nowadays, ground-coupled heat pump system (GCHP) becomes one of the most energy-efficient systems in heating, cooling and hot water supply. However, it remains challenging to accurately predict thermal energy conversion, and the numerical calculation methods are too complicated. First, according to seasonality, this paper analyzes four variables, including the power consumption of heat pump, the power consumption of system, the ratios of the heating capacity (or the refrigerating capacity) of heat pump to the operating powers of heat pump and to the total system, respectively. Then, heat transfer performance of GCHP by historical data and working parameters is predicted by using random forests algorithm based on autoregressive model and introducing working parameters. Finally, we conduct experiments on 360-months (30-years) data generated by GCHP software. Among them, the first 300 months of data are used for training the model, and the last 60 months of data are used for prediction. Benefitting from the working condition inputs it contained, our model achieves lower Mean Absolute Error (MAE), Mean Absolute Percentage Error (MAPE), Root Mean Square Error (RMSE) than Exponential Smoothing (ES), Autoregressive Model (AR), Autoregressive Moving Average Model (ARMA) and Auto-regressive Integrated Moving Average Model (ARIMA) without working condition inputs.

## Introduction

The ground-coupled heat pump system (GCHP) system (Tian, Wu & Shi, 2016) is an energy utilization system to employ environment-friendly, pollution-free alternative energy source, addressing the challenge of balancing the resource consumption (such as mineral resources) with clean environment. Compared with common resources, heat pumps have the following advantages: (a) significant environmental and economic benefits, (b) no pollution or squander of water source, (c) extensive applications in air-conditioning, refrigeration, heating, and daily hot water supply, etc., (d) low maintenance cost, guard-free and space saving. Evelyn et al. (2020) simulated five occupants residential dwelling in a largest village in Nunavik, a very subarctic remote Canada region, and they verified that shallow geothermal energy through state of-the-art heat pumps is the most economically attractive heating option. Because of its sustainable development ability, GCHP system becomes more and more popular all over the world.

The performance of the GCHP system may degrade when installed in heating-dominated buildings, where the amount of heat extracted from the ground exceeds that of the released. Therefore, the total heat loss may reduce the temperature of intake water. This thermal imbalance can be eliminated by increasing the space between boreholes, as well as by adopting strip type and block layout with higher-level initial investment (Yang, Chen & Shi, 2013). Other methods are also developed to address this problem. Trillat-Berdal, Souyri & Fraisse (2006) presented an experimental study of a GCHP combined with thermal solar collectors, whose advantages are the balance of the ground loads, longer operating time of the solar collectors and avoidance of overheating. Several alternative heat compensations have been studied to date. Tian et al. (2017) proposed GCHP with a coupled operation of the heat compensation unit to investigate such issues as heating capacity deficiency at peak heating loads and high borehole investment, and they found that during a heating season, the temperature of the fluid entering the evaporator of heat pump can be increased by the heat compensation unit. By comparison with a non-coupled system, they built a TRNSYS model for evaluation of the system reliability, efficiency and economy, and they came to the conclusion that the coupled system’s payback period was only 1 year. In view of the limited accessible land area, plate ground heat exchanger (GHE) is paid more attention to due to its highest heat transfer rate per unit. Therefore, Ali, Mohammad & Ali (2020) made a profound study of the thermal performance of vertical plate GHEs caused by GHE spacing, buried depth, the height of GHE, soil type, and so on. They found that avoiding the adverse effect of thermal interference, 4 m is the optimum distance between two adjacent GHEs through their built 3-D numerical model. Linfeng et al. (2018) proposed a novel hourly simulation method for the energy performance of an office building served by a GCHP system. In their method, Fast Fourier Transform (FFT) was used to analyze the coefficient of performance (COP) of the system because of its computation speed and accuracy. Furthermore, U-pipe shank spacing under various models, fluid and ground temperature on GCHP system performance are also analyzed.

The efficiency, cost-effectiveness, and durability of the heat pump whether it can be accepted and deployed in any new building heating and/or cooling technology. In order to assess building energy performance, researchers have worked with various data mining techniques such as adaptive neuro-fuzzy inference systems (ANFIS) (Esen, Inalli & Sengur, 2008a), support vector machine (SVM) (Esen, Inalli & Sengur, 2008c), iteratively reweighed least squared (IRLS) (Tsanas & Xifara, 2012), random forest (RF) (Tsanas & Xifara, 2012), multiple regression model (Catalina, Iordache & Caracaleanu, 2013), multivariate adaptive regression splines (MARS) (Cheng & Cao, 2014) and artificial neural network (ANN) (Sun, Hu & Lei, 2015). In our previous work (Zhuang, Ben & Yan, 2017), M5 Model Tree, Support Vector Regression (SVR) and Partial Least Squares Regression (PLSR) are used to predict the heat transfer performance for the GCHP system. However, these methods only consider the time series and ignore the influence of working condition inputs, and there has been few works on predicting the performance of solar-assisted ground-coupled heat pump systems.

In order to make full use of both working condition information and history data and make up for the lack of work on solar-assisted GCHP system, we propose a novel autoregressive model with working condition inputs to accurately predicting heat transfer performance of solar-assisted ground-coupled heat pump system.

Our main contributions of this paper are summarized as follows. First, we find the seasonal factor decomposition is good for predicting heat transfer performance of ground coupled heat pump system. Second, working condition inputs are introduced to the prediction model, which can achieve lower MAE, MAPE, RMSE than ES, AR, ARMA and ARIMA without working condition inputs. Third, our code and model are available at https://github.com/JayShaun/ARX.

### Related work

With the flourish of machine learning and big data technology, some data-mining based methods, such as ANN and SVM have been applied to predict the energy transfer performance. Zhao, Zhong & Zhang (2016) applied ANN and SVM models to do energy consumption predicting of variable refrigerant volume (VRV) system in office buildings. Le Cam, Daoud & Zmeureanu (2016) presented an application of the process of knowledge discovery in databases (KDD) using nonlinear ANN for the forecasting of the electrical power demand of a supply fan of an AHU (air handling unit). Ceci, Corizzo & Fumarola (2017) tackled the problem of power prediction of several photovoltaic (PV) plants and indicated that regression trees provided better models than artificial neural networks on two PV power plant datasets. Ceci et al. (2020) also proposed an unsupervised change detection method which is able to analyze streaming data generated by sensors located in smart grid. Yan, Hu & Li (2016) studied the performance prediction of ground source heat pump (GSHP) systems by real-time monitoring data and data-driven models. They used back-propagation neural network (BPNN) algorithm to establish the data-driven models. Candanedo, Feldheim & Deramaix (2017) presented and discussed data-driven predictive models for the energy use of appliances. They trained four statistical models: multiple linear regression, support vector machine with radial kernel, random forest and gradient boosting machines (GBM) to predict energy used in a low-energy house. Xiao, Wang & Cheng (2017) explored the potential application of advanced data mining techniques for effective utilization of big building operational data. Deep learning-based prediction techniques, decision tree and association rule mining were adopted to analyze the operational data. Bedi & Toshniwal (2018) proposed an empirical mode decomposition method combined with the long short-term memory network to estimate electricity demand for the given season, day, and time interval of a day. Mathioulakis, Panaras & Belessiotis (2018) introduced artificial neural networks for the performance prediction of heat pump hot water heaters, which verified that a trained ANN could represent an effective tool for the prediction of the air-to-water heat pump (AWHP) performance in various operation conditions and the parametrical investigation of their behavior. Xia, Ma & Kokogiannakis (2018) presented a model-based design optimization strategy for ground source heat pump systems with integrated solar photovoltaic thermal collectors (GSHP-PVT). The highlight of this paper was an artificial neural network (ANN) model was used for performance prediction and a genetic algorithm (GA) was implemented as the optimization technique.

Some data mining methods were also applied to forecast the performance of ground-coupled heat pump system. Esen, Inalli & Sengur (2008b) tried to improve the performance of an artificial neural network with a statistical weighted pre-processing method to learn to predict ground source heat pump systems with the minimum data set. They used three work conditions as input layer, while the output was coefficient of performance of system. Besides, they reported on a modeling study of ground coupled heat pump system performance by using a support vector machine method (Esen, Inalli & Sengur, 2008d). Wang et al. (2013) proposed an application of artificial neural networks based on improved Radial Basis Function (NNCA-RBF) to predict performance of a horizontal ground-coupled heat pump system. Roberto et al. (2021) propose a Tucker tensor decomposition, capable of extracting a new feature space for forecasting the multiaspect renewable energy.

These mentioned methods only use either time series information to make a time series prediction or external inputs to simulate a regression function. This paper proposes an autoregressive model with working condition inputs to accurately predicting heat transfer performance of ground-coupled heat pump system.

### Data

A quantitative validation of heat transfer performance data analysis has been obtained according to GCHP software, which is developed by ZhongRui New Energy Science & Technology Co., Ltd. 5,000 working conditions of solar-assisted GCHP system with vertical GHE during 30 years (360 months) is simulated, and the detailed description of the input data (working conditions variables) can be found in our previous work (Zhuang, Ben & Yan, 2017).

### Working conditions variables

The 30 years’ heat transfer performance data including heat exchange ability, temperature of circulating liquid, inlet and outlet temperature of heat pump are simulated. Table 1 shows the detailed characteristics of input parameters. In Table 1, numerical types (Type for short) of the data include real and nominal numbers. The minimum (Min. for short) and maximum (Max. for short) are the smallest and largest values of a set of an input variable in 5,000 working conditions, respectively. The average (Avrg. for short) is the mean value of a set of an input variable in 5,000 working conditions, denoting a measure of central tendency. Standard deviation (Std. for short) is a measure used to quantify the amount of variation or dispersion of a set of data values. Range measures the difference between the Max. and Min. in a set. The median (Med. for short) can be found by arranging all the observations from lowest value to highest value and picking the middle one.

**Table 1:** Input parameters.

Variable	Type	Min.	Max.	Avrg.	Std.	Range	Med
*X*_1_-Borehole arrangement	Nominal	1	5	3	1.4144	4	3
*X*_2_-Borehole depth (mm)	Real	100,000	120,000	109,950	8,155.4	20,000	110,000
*X*_3_-Borehole radius (mm)	Real	55	75	65	7.1	20	65
*X*_4_-Borehole number	Real	46	56	50.956	3.9546	10	50
*X*_5_-Vertical spacing (mm)	Real	4,000	5,000	4,495	409.3	1000	4,500
*X*_6_-Column spacing (mm)	Real	4,000	5,000	4,478	410.2	1,000	4,500
*X*_7_-Thermal conductivity coefficient (W/(mm ⋅ °C))	Real	0.0013	0.0028	0.002049	0.000461	0.0015	0.002
*X*_8_-Nominal external diameter (mm)	Real	1,000	2,000	1,499	500	1,000	1,000
*X*_9_-U-tube spacing	Nominal	1	4	2.498	1.1189	3	2
*X*_10_-Temperature (°C)	Real	10	20	14.997	3.1624	10	15
*X*_11_-Thermal conductivity	Nominal	1	8	4.416	2.253	7	4
*X*_12_-Circulating liquid parameter	Nominal	1	11	5.971	3.1683	10	6

### Heat pump performance output data

**Figure 1:**
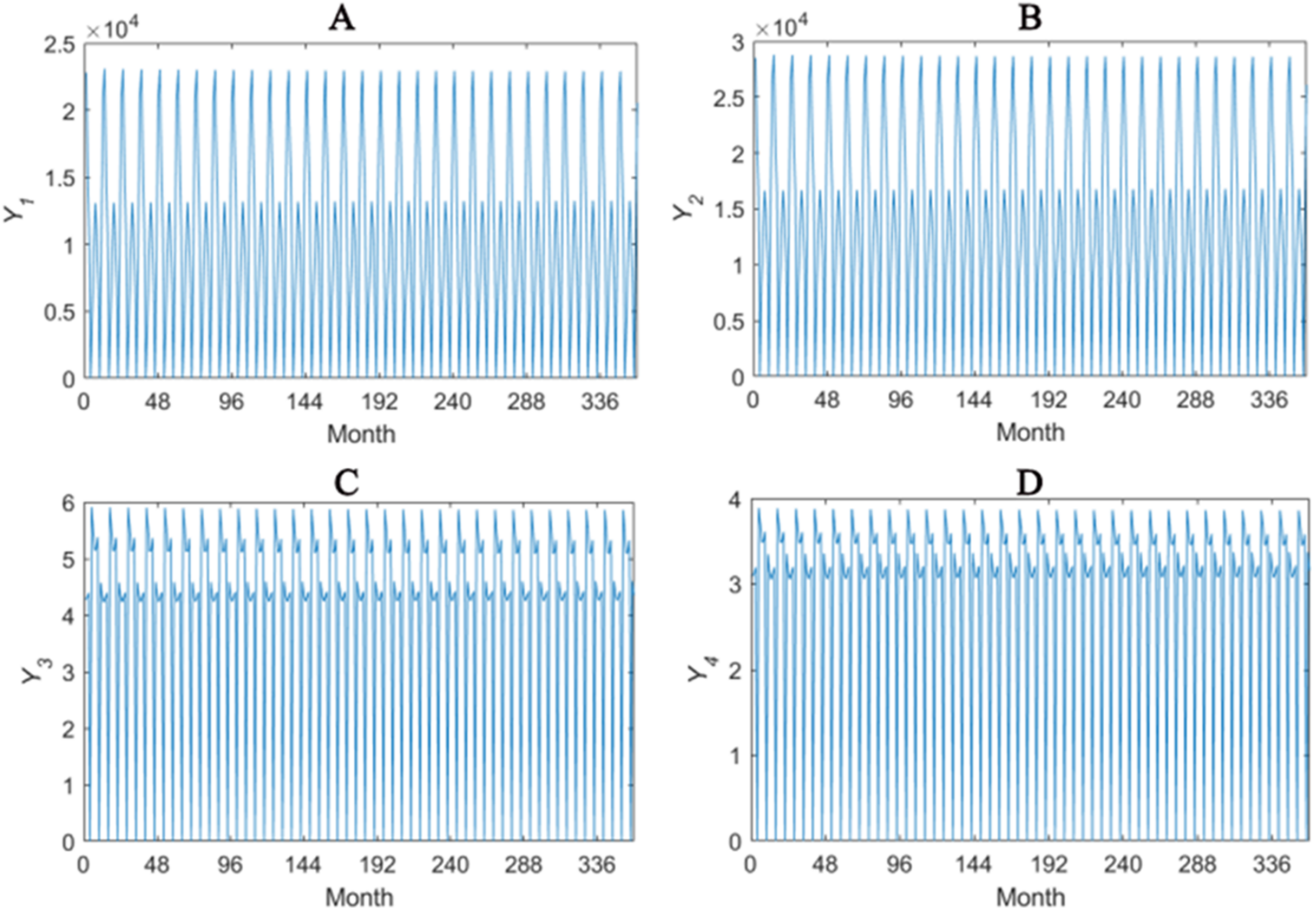
Overall tendencies of Y_1_–Y_4_ (A–D).

The solar-assisted GCHP system for 360 months are simulated and the heat transfer performance *Y*_1_–*Y*_4_ are recorded. *Y*_1_ denotes the monthly power consumption of heat pump. *Y*_2_ denotes the monthly power consumption of whole system, including heat pump and water pump. *Y*_3_ denotes the ratio of the heating capacity or the refrigerating capacity of heat pump to the operating power of heat pump. *Y*_4_ denotes the ratio of the heating capacity or the refrigerating capacity of heat pump to the total operating power of system. The overall tendencies of *Y*_1_–*Y*_4_ are given in Fig. 1.

## Methodology

In this section, seasonal factor decomposition and Autoregressive model with working condition inputs are described in detail.

### Seasonal factor decomposition

The relevant temperature data, strongly influenced by the weather, is a seasonal periodical time series. Generally speaking, seasonal time series have the following components: tendency (*T*), cycle (*C*), seasonality (*S*), irregular variations (*I*). Additive seasonal decomposition model can be expressed as: *Y*_*t*_ = *T*_*t*_ + *S*_*t*_ + *C*_*t*_ + *I*_*t*_ and a term-by-term separation is as follows:

#### Trend item: moving average prediction method

The observed value of time series is denoted as *y*_*t*_,*t* = 1,2,…,*N*, and the moving average value is calculated as follows:

(1) $$M_{t}=\frac{y_{t}+y_{t-1}+\cdots +y_{t-n-1}}{n},\;t=1,2,...,N,\;n⩾2.$$

Consequently, the predicted value for *t* + 1 would be ${\hat{y}}_{t+1}=M_{t}$, therefore, the time series without trend term is obtained.

#### Seasonal factor item: trend extrapolation separation prediction method

Firstly, assuming that the time series contains no trend item, its model can be written as:

(2) Y=S+C+I.

Since the effect of periodical component *C* is subtle, it can be integrated into stochastic error component *I*. When separating the seasonal factor item, the periodical item and stochastic term can be viewed as a whole, and the stochastic error component is major. Then, the monthly average of *Y* is calculated, and after subtraction, the seasonal factor item *S* without stochastic error component is obtained.

#### Cycle item: periodogram method

Assuming that time series contains neither trend term nor seasonal term. Firstly, it is decentralized as:

(3) $$x_{i}^{\prime }=x_{i}-\frac{1}{N}\sum_{i=1}^{N}x_{i}.$$

Then it can be written in the forms of Fourier series. *A*_*τ*_, *B*_*τ*_ denote Fourier coefficients, *τ* as the period, and it is set that $S_{\tau }^{2}=A_{\tau }^{2}+B_{\tau }^{2}$ and there will be a periodical change in the data. The concrete computation process is as follows: the variance $\sigma_{x}^{2}$ of *x*_*i*_ is calculated to investigate that $S_{\tau }^{2}$ is the largest value of the periodical vibration. Amplitude and phase are formulated as $C_{\tau }=\sqrt{A_{\tau }^{2}+B_{\tau }^{2}}$, $\phi_{\tau }=\arctan\frac{A_{\tau }}{B_{\tau }}$. Therefore, the periodic vibration can be formulated ${\hat{C}}_{1}(t)=C_{t}\sin\left(\frac{2\pi t}{\tau }+\phi_{t}\right)$. Moreover, multiple period calculations can be performed.

#### Stochastic error item

After having separated trend item, seasonal factor item and cycle item from the series, what is left is the stochastic error item.

### Autoregressive model with working condition inputs

For an output variable *Y*, a time serie can be defined as $\left\{y_{t},t=1,2,...,T\right\}$, under a working condition parameter ***x***. *T* denotes the length of time series, and ***x*** denotes a *d*-dimensional vector of working condition inputs. The aim is to construct the appropriate model and to predict its future values by using historical data of *Y* as well as these working condition parameters (*X*_1_–*X*_12_).

The predictive value ${\hat{y}}_{t}$ for the moment *t* can be obtained by general form of autoregressive (AR) model:

(4) $${\hat{y}}_{t}=f(y_{t-1},y_{t-2},...,y_{t-M}),$$

where *M* is a lagged term. Therefore (4) indicates that the historical data of *Y* can predict its future values. Furthermore, these exogenous variables such as working condition parameters to the autoregressive (AR) model is introduced, therefore

(5) $${\hat{y}}_{t}=f(x,y_{t-1},y_{t-2},...,y_{t-M}).$$

Formula (5) indicates that the future values ${\hat{y}}_{t}$ are predicted by not only the historical data of *Y* but also the working condition parameters and this is autoregressive model with working condition inputs.

The next step is to construct Random Forests according to the data of the working conditions and time series. Random Forests are constituted by Classification And Regression Tree (CART). Firstly, *m* attributes from all (*d* + 1) × *M* + *d* attributes are randomly extracted and the optimal attributes should be selected from *m* attributes to be split. Then the subtrees are generated iteratively until attributes cannot be split. Ultimately, a regression tree is formed. The optimal split property selection is based on the minimum variance of the two sub-nodes that the property is divided into. In terms of attribute set *A* of a note, one of the attributes (*a*) is regarded as split attribute to split into two nodes *D*_1_, *D*_2_, and the optimal split attribute is as follows:

(6) $$a^{*}=\arg\underset{a\in A}{\min}[V(D_{1})+V(D_{2})],$$

where *V*(*D*_1_), *V*(*D*_2_) are variances of *D*_1_, *D*_2_ respectively.

By using the method described above, *K* classification and regression trees are generated, then the random forest is constructed. The average of regression *K* results is the predicted value for the moment *t*.

(7) $${\hat{y}}_{t}=\frac{1}{K}\sum_{k=1}^{K}f_{k}(x,y_{t-1},y_{t-2},...,y_{t-M}),$$

where *f*_*k*_(·) denotes the regression result of a regression tree *k*.

## Experimental tests

In this section, seasonal factor decomposition and autoregressive model with working condition inputs (introduced in “Methodology”) are used to conduct the experiments on the data of solar-assisted GCHP system (introduced in “Data”). In particular, the sensitive parameter of autoregressive model with working condition inputs is the only lagged term *M*. Since our data characteristics are approximately annual periodic changes, the value of *M* is related to the period obtained by the seasonal factor decomposition.

### Error measurements

Three error indicators including Mean Absolute Error (MAE), Mean Absolute Percentage Error (MAPE) and Root Mean Square Error (RMSE) are used in this paper to measure the prediction accuracy. *N* donates the number of time series and *T* denotes the length of a time series predicted. ${\hat{y}}_{t}^{\left(i\right)}$ and $y_{t}^{\left(i\right)}$ donates the predicted value and the observed value for the moment *t* of the *i*-th time series, respectively.

(8) $$MAE=\frac{1}{TN}\sum_{t=1}^{T}\sum_{i=1}^{N}|{\hat{y}}_{t}^{\left(i\right)}-y_{t}^{\left(i\right)}|$$

(9) $$MAPE=\frac{100}{TN}\sum_{t=1}^{T}\sum_{i=1}^{N}\left|\frac{{\hat{y}}_{t}^{\left(i\right)}-y_{t}^{\left(i\right)}}{y_{t}^{\left(i\right)}}\right|$$

(10) $$RMSE=\sqrt{\frac{1}{TN}\sum_{t=1}^{T}\sum_{i=1}^{N}{\left({\hat{y}}_{t}^{\left(i\right)}-y_{t}^{\left(i\right)}\right)}^{2}}$$

MAE, which is the rate of absolute value of difference between predicted value and observed value and the number of predicted data, reflects the average distance of predicted value’s deviation. MAPE is another accuracy measurement in statistics and indicates the proportion of predicted value in observed value, reflecting the relative error. RMSE is square root of ratio, which can be calculated by square sum of difference between predicted value and observed value and the number of predicted data. RMSE indicates the extent of deviation and is sensitive to particularly large or particularly small errors in predicted values.

### Seasonal factor decomposition results and analysis

**Figure 2:**
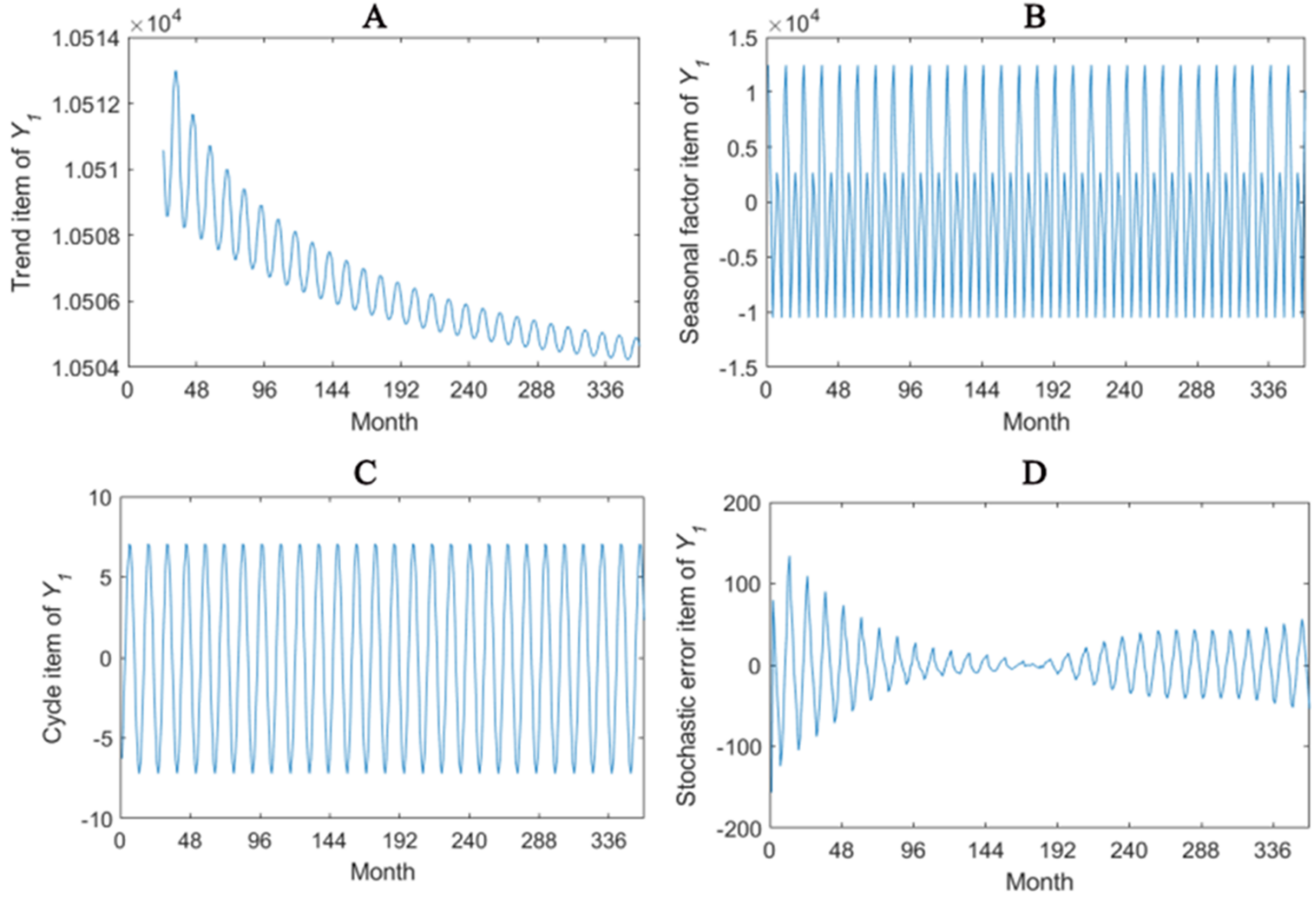
Seasonal factor decomposition of Y_1_. (A) Trend item; (B) Seasonal factor; (C) Cyber item; (D) Stochastic error item.

**Figure 3:**
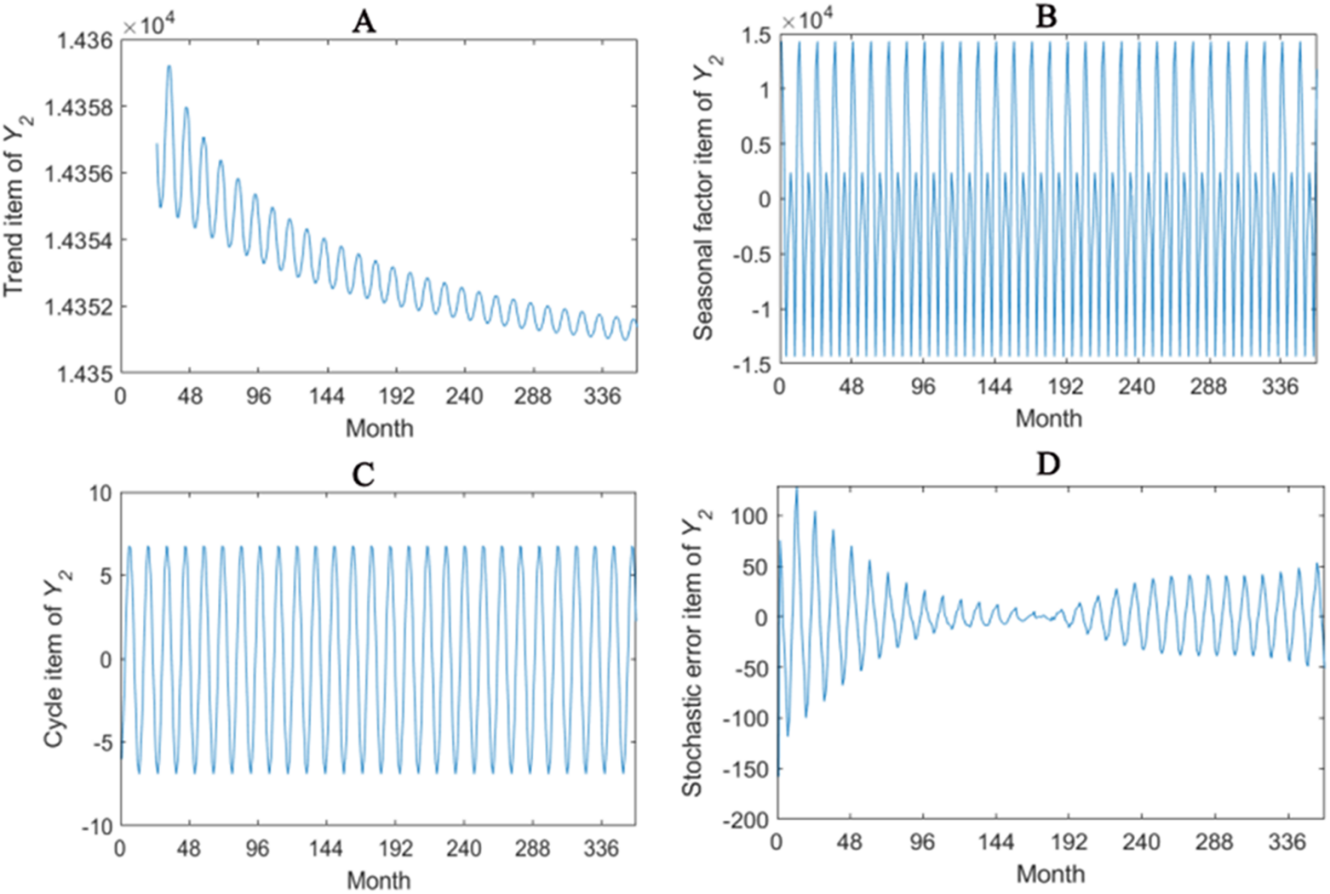
Seasonal factor decomposition of Y_2_. (A) Trend item; (B) Seasonal factor; (C) Cyber item; (D) Stochastic error item.

**Figure 4:**
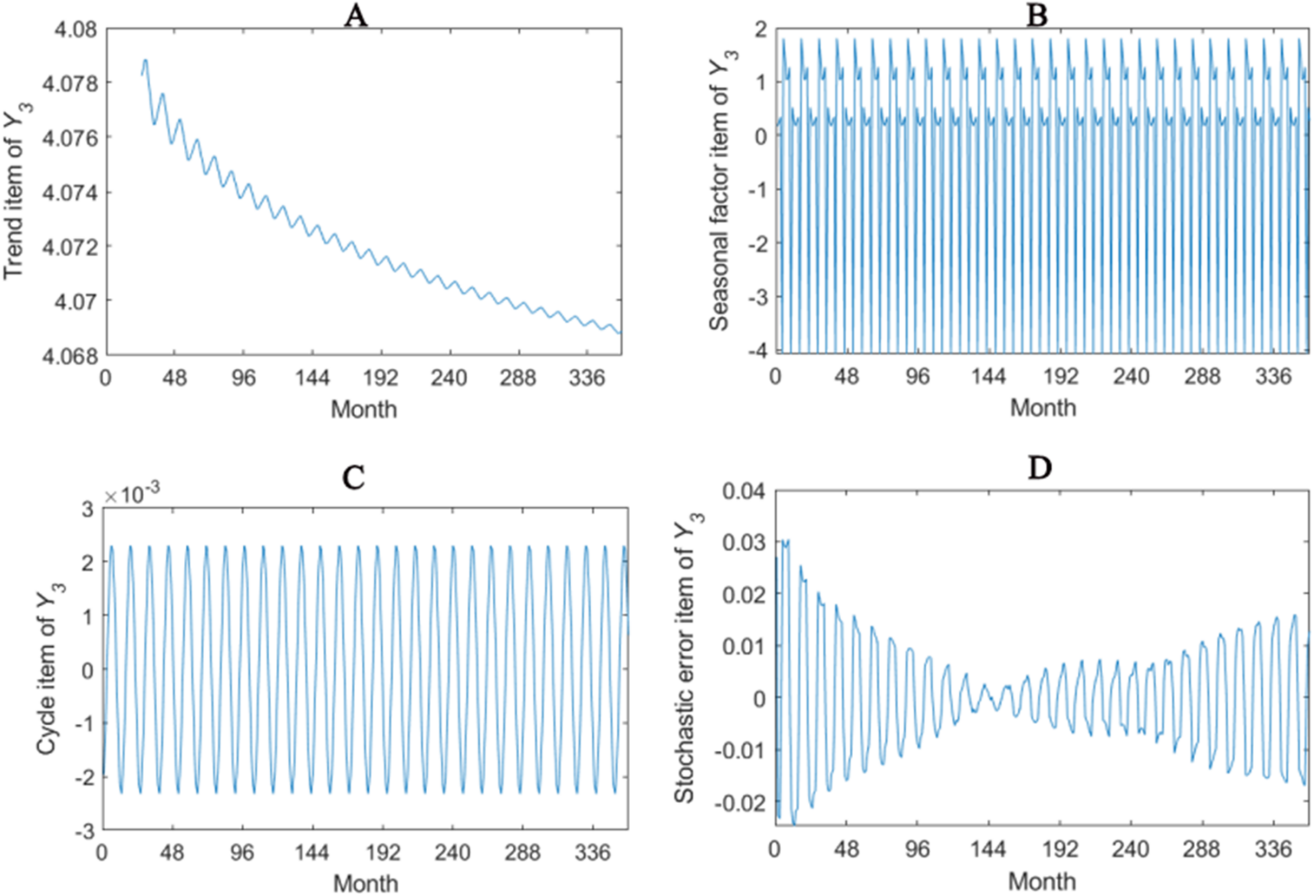
Seasonal factor decomposition of Y_3_. (A) Trend item; (B) Seasonal factor; (C) Cyber item; (D) Stochastic error item.

**Figure 5:**
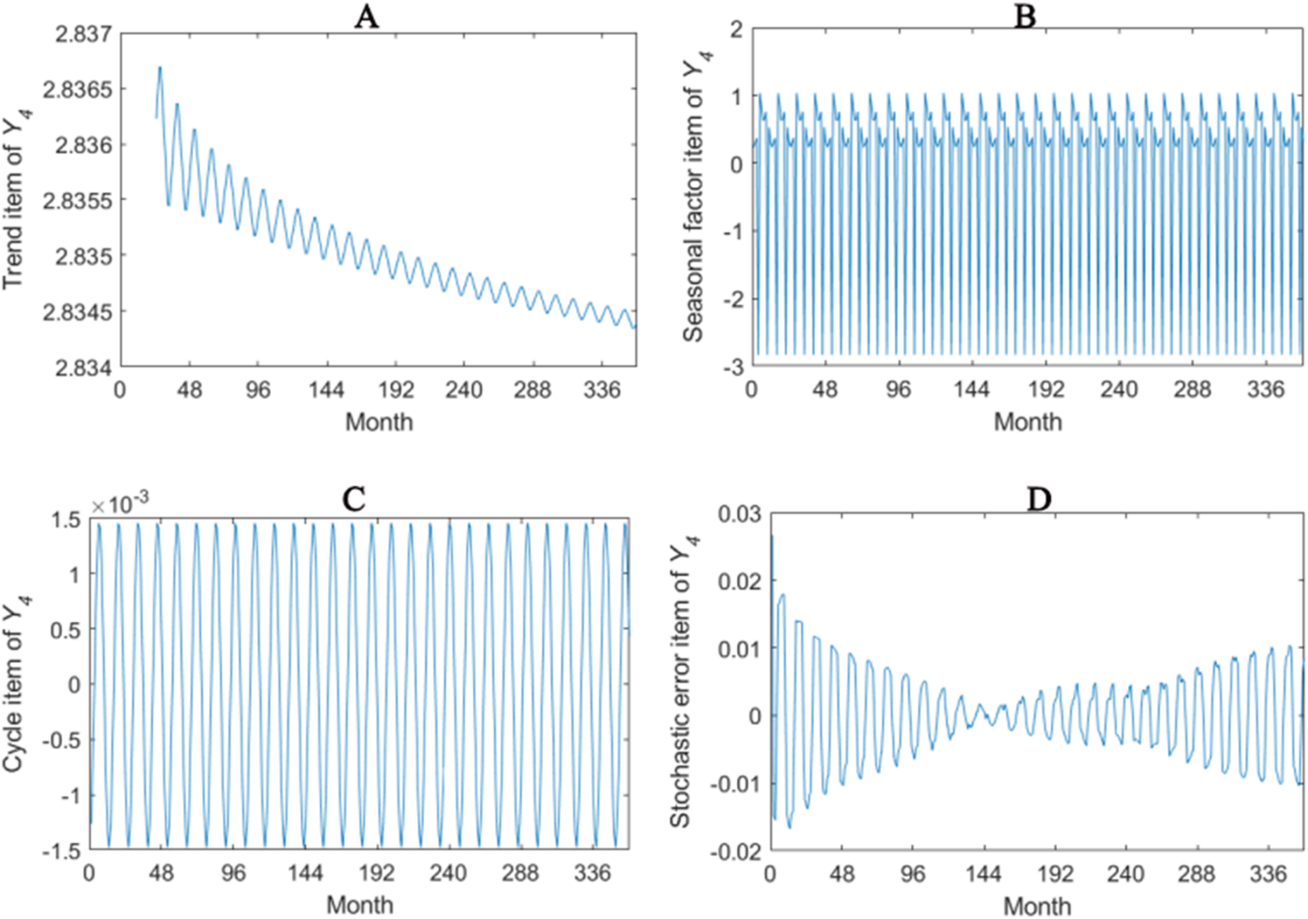
Seasonal factor decomposition of Y_4_. (A) Trend item; (B) Seasonal factor; (C) Cyber item; (D) Stochastic error item.

The trend item, seasonal factor itemcycle item and stochastic error item for *Y*_1_–*Y*_4_ are given in Figs. 2–5. As for *Y*_1_, the trend item shows a considerably downward trend during the period. From seasonal items it has obvious seasonal characteristic. In practical terms, the power consumption of heat pump drops year by year, while the working state of it changes with different seasons. The cycle item of implies that the time series is periodical and it can be seen that its value is small and its influence is weak. Stochastic error item reflects some uncertain factors of series. It has a small amount of fluctuation with 0, and the effect is very small. *Y*_2_, *Y*_3_ and *Y*_4_ have similar features with *Y*_1_. The periods of *Y*_1_, *Y*_2_, *Y*_3_ and *Y*_4_ are all 12 months.

### Time series analysis results and analysis

In time series analysis, there are 5,000 series in *Y*_1_, *Y*_2_, *Y*_3_ and *Y*_4_ respectively and each series consists of 360-month data, which means 5,000 × 360 dimensions. For the prediction of the value in future 60 months, the model is trained by the historical data of the first 300 months. We trained our model i.e. autoregressive model with working condition inputs (ARX for short) at each time step by using random-forest library in Matlab. It is note that we utilize the earlier predicting results as known data to predict later values at test stage. Combined with the results of seasonal decomposition of output data in “Seasonal Factor Decomposition Results and Analysis”, the lagged term in regression model should be set to an integral multiple of one period. If the setting is too small (i.e., one period (12 month)), the regular pattern of time series is not clearly caught. On the contrary, if the setting is too large, the number of random trees participating in the calculation will increase, which makes the model more complex. Therefore, the value of the lagged term *M* should be more than one period, and we tested two smaller values (*M* = 24, and *M* =3 6) in the ARX model. Table 2 lists the MAE results. The lower MAEs are obtains under *M* = 24. So the lagged term in regression model is set as 24 months. The sliding window is same as lagged term which is 24. We compare several time series analysis methods, including Exponential Smoothing (ES), Autoregressive Model (AR), Autoregressive Moving Average Model (ARMA) and Auto-regressive Integrated Moving Average Model (ARIMA). Exponential Smoothing (ES) is one of moving average methods which is characterized by applying different weights to the observed values of the past. The weight of short-term observed value is greater than the long-term. The basic idea is that predicted value is a weighted sum of the observed values with different weights (new data with greater weight). ARIMA regards the data series formed by forecasting object with the change of time as the random series and sets certain model to approximately describe this series. Once the model is recognized, the time series’ past value and present value can be applied to forecast its future value. Similarly, AR and ARMA can be set according to ARIMA. In detail, the values of both difference and moving average are 0 in AR, while only the value of difference is 0 in ARMA. The smoothing factor of ES is 0.1. For ARIMA, the auto-regressive *p*, the integrated term (differential order) *d* and the moving average lag *q* are set as 12, 1 and 12, respectively. And the parameters of the others methods is same with ARIMA, i.e., AR (*p* = 12) and ARMA (*p* = 12, *q* = 12).

**Table 2:** Prediction Results (average ± std.) of ARX under different values of the lagged term *M*.

*M*	24	36
*Y*_1_	1.4563 *±* 1.0675	1.4948 *±* 1.0972
*Y*_2_	1.5841 *±* 1.0675	1.6478 *±* 1.3396
*Y*_3_	0.0005 *±* 0.0004	0.0007 *±* 0.0005
*Y*_4_	0.0004 *±* 0.0002	0.0006 *±* 0.0003

In order to measure overview errors of the predicted 60-month value, we average 60-month errors of *Y*_1_, *Y*_2_, *Y*_3_ and *Y*_4_ respectively (Table 3). Compared with ES, AR, ARMA, ARIMA, Autoregressive model with working condition inputs (ARX) has prominent advantage with smallest mean absolute error, mean absolute percentage error and root mean square error. In “Seasonal Factor Decomposition Results and Analysis”, according to decomposition of seasonality, four outputs are stationary time series. Therefore, the large error can be witnessed in moving average method, which is the reason of low precision in ES. ARX added 12 working condition parameters to predict values, which lack in traditional time series analysis. The results of experiment show that adding working parameters can improve the predicted precision of time series. This is because that four output variables including the power consumption of heat pump, the power consumption of system, the ratios of the heating capacity (or the refrigerating capacity) of heat pump to the operating powers of heat pump and to the total system, respectively show different tendencies under various working parameters. In other words, it is limited to simply use historical data to predict the future values and ignore the influence of system parameters, such as hole, u-tube and collector. In practical working situation, working states of the system are definitely different with different parameters. The prediction of ignoring these parameters is only general and does not take the particularity of different systems into account. In terms of model, the introduction of these additional parameters has a similar effect on the prediction model with a window limit. Prediction result cannot deviate from its output restrictions of particular parameters. Ultimately, the prediction curve will be adjusted in a better direction.

**Table 3:** Prediction results (average ± std).

		ES	AR	ARMA	ARIMA	ARX
*Y*_1_	MAE	3.2474 *±* 3.6914	4.4499 *±* 4.0379	4.0180 *±* 4.4986	1.6077 *±* 1.9051	**1.4563 *±* 1.0675**
	MAPE	0.0201 *±* 0.0197	0.0409 *±* 0.0396	0.0263 *±* 0.0235	0.0136 *±* 0.0121	**0.0134 *±* 0.0105**
	RMSE	4.6730	11.2387	6.2574	5.9016	**3.3524**
*Y*_2_	MAE	3.6285 *±* 3.0647	4.8445 *±* 4.0373	4.1315 *±* 4.1888	3.8640 *±* 3.9055	**1.5841 *±* 1.0675**
	MAPE	0.0218 *±* 0.0203	0.0323 *±* 0.0309	0.0232 *±* 0.0219	0.0209 *±* 0.0197	**0.0105 *±* 0.0094**
	RMSE	4.4828	11.7774	6.7255	5.2054	**3.5126**
*Y*_3_	MAE	0.0022 *±* 0.0011	0.0016 *±* 0.0012	0.0022 *±* 0.0009	0.0022 *±* 0.0007	**0.0005 *±* 0.0004**
	MAPE	0.0530 *±* 0.0516	0.0409 *±* 0.0379	0.0530 *±* 0.0492	0.0503 *±* 0.0482	**0.0134 *±* 0.0122**
	RMSE	0.0026	0.0035	0.0026	0.0026	**0.0011**
*Y*_4_	MAE	0.0021 *±* 0.0004	0.0011 *±* 0.0005	0.0020 *±* 0.0005	0.0020 *±* 0.0004	**0.0004 *±* 0.0002**
	MAPE	0.0750 *±* 0.0732	0.0425 *±* 0.0417	0.0727 *±* 0.0716	0.0727 *±* 0.0715	**0.0138 *±* 0.0131**
	RMSE	0.0026	0.0024	0.0025	0.0025	**0.0008**

**Note:**

Best results are in bold.

**Figure 6:**
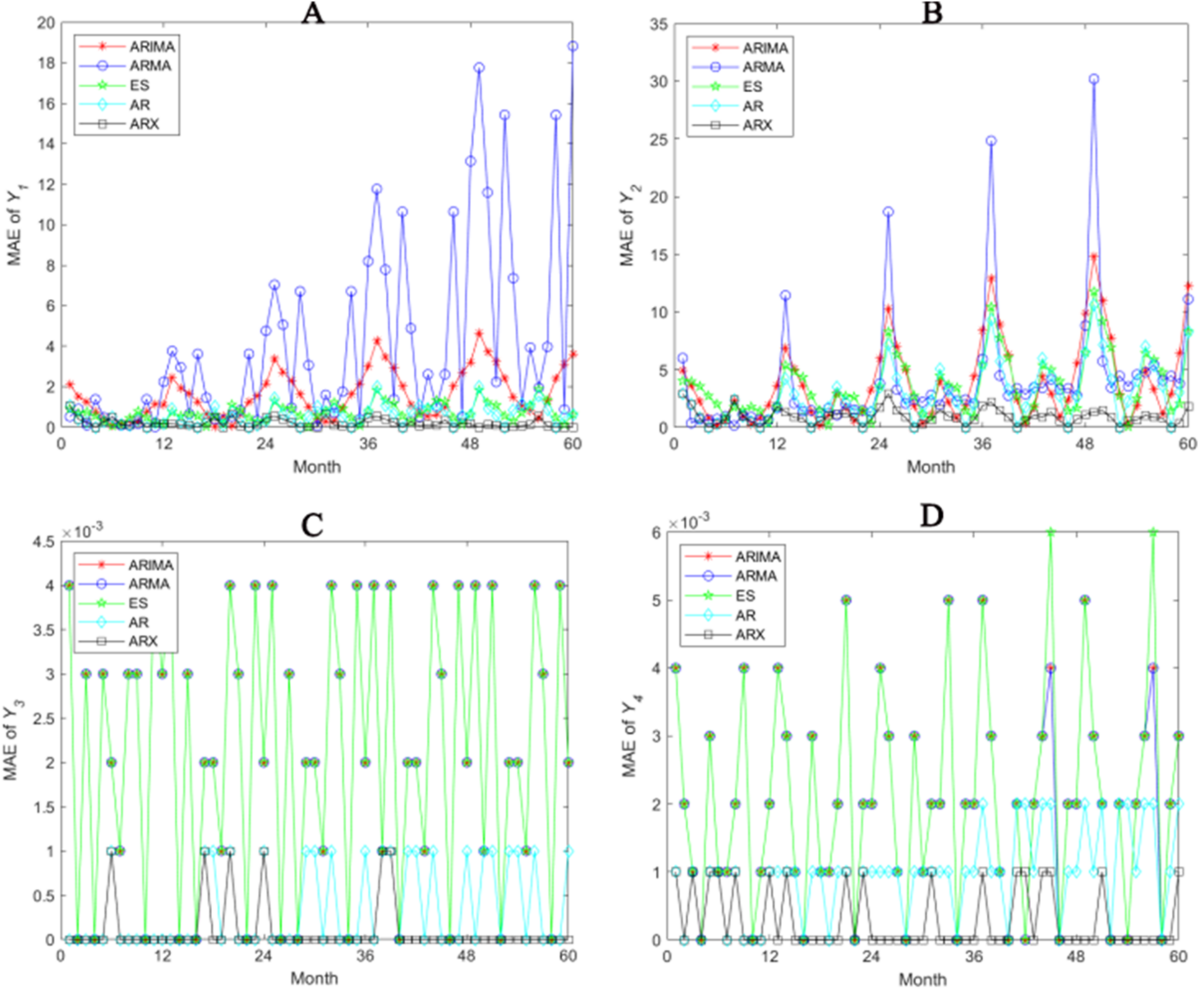
Prediction accuracies of different methods (A–D).

In order to prove the positive effects of our prediction model, we draw the error graphs of *Y*_1_, *Y*_2_, *Y*_3_ and *Y*_4_ in 60 months with using MAE as an example respectively (see Fig. 6). First of all, obvious periodicity can be witnessed in MAE, periodic value of which is 12 (1 year), meeting the periodical requirement of practical system. In addition to the ES method, prediction accuracy shows a downward trend because the model predictions of heat pump and system’s electricity consumption become inaccurate as time goes on. As for this problem, the Autoregressive model with working condition inputs model well embodies the adjustment function of the external parameters, though not completely eliminate the accuracy decreasing trend, but the performance is much better. Taking seasonality into consideration, we find that the working state of heat pump in every month is regular. Therefore, between-year is used to predict, which means the prediction of a month is based on the data of the same month over the past years. In practice, the system is closed in April and October without electricity consumption. The predictions of these 2 months are not 0 in ARIMA, so the model is unrealistic. Nevertheless, Autoregressive model with working condition inputs has no prediction error in these months is in accordance with the practical circumstances. In spite of introducing extra parameters, the between-year-based model avoids deviating prediction from practical situation.

## Conclusion

Ground-coupled heat pump system (GCHP) uses surficial energy storage to provides different kinds of structures with efficient heating, cooling and hot water supply so as to reduce the consumption of electric and gas. GCHP, a kind of alternative energy in heating and cooling, plays a significant role in residence and commerce. The thermal energy conversion of GCHP is predicted by Autoregressive model with working condition inputs. Experimental results show that working parameters can improve prediction accuracy of four output variables, including the power consumption of heat pump *Y*_1_, the power consumption of system *Y*_2_, the ratio of the heating capacity (or the refrigerating capacity) of heat pump to the operating powers of heat pump *Y*_3_ and the ratio of the heating capacity (or the refrigerating capacity) of heat pump to to the total system *Y*_4_. In comparison to traditional time series model (such as AR), we can not only take full advantage of given working condition inputs information, but also can set specific model in a time series with certain parameters because different time series has different working parameters. In the future, the measure based on time series analysis can be combined with learning algorithms (such as Gradient Boost, metric learning).

## Additional Information and Declarations

### Data Availability

The following information was supplied regarding data availability: The MATLAB code is available at GitHub: https://github.com/JayShaun/ARX.
